# Phenotypic stratification and genotype–phenotype correlation in a heterogeneous, international cohort of GNE myopathy patients: First report from the GNE myopathy Disease Monitoring Program, registry portion

**DOI:** 10.1016/j.nmd.2017.11.001

**Published:** 2018-02

**Authors:** Oksana Pogoryelova, Phillip Cammish, Hank Mansbach, Zohar Argov, Ichizo Nishino, Alison Skrinar, Yiumo Chan, Shahriar Nafissi, Hosein Shamshiri, Emil Kakkis, Hanns Lochmüller

**Affiliations:** a The John Walton Muscular Dystrophy Research Centre, Newcastle University, UK; b Ultragenyx Pharmaceutical Inc. Novato, CA, USA; c Department of Neurology, Hadassah-Hebrew University Medical Center, Jerusalem, Israel; d Department of Neuromuscular Research, National Institute of Neuroscience, National Center of Neurology and Psychiatry, Tokyo, Japan; e Department of Neurology, Tehran University of Medical Sciences, Tehran, Iran

**Keywords:** GNE myopathy, Hereditary inclusion body myopathy, Distal myopathy, Rare neuromuscular disorders, Epidemiology, Genotype-phenotype correlation

## Abstract

GNE myopathy is a rare distal myopathy, caused by mutations in the GNE gene, affecting sialic acid synthesis. Clinical presentation varies from asymptomatic early stage patients to severely debilitating forms. This first report describes clinical presentations and severity of the disease, using data of 150 patients collected via the on-line, patient-reported registry component of the GNE Myopathy Disease Monitoring Program (GNEM-DMP). Disease progression was prospectively analysed, over a 2-year period, using the GNE myopathy functional activity scale (GNEM-FAS). The average annual rates of decline in function were estimated at −9.6% and −3.2% in ambulant and non-ambulant patients respectively. 4.3% of participants became non-ambulant within one year. The mean time from onset to required use of a wheelchair was 11.9 years. Mean delay of genetic diagnosis from symptom onset was 5.2 years. Mutation specific analysis demonstrated genotype–phenotype relationships; i.e. p.Ala662Val may be associated with a more severe phenotype, compared to p.Val727Met. Patients with compound heterozygous mutation in epimerase and kinase domain appeared to have a more severe phenotype compared to patients with both mutations located within one domain. Acknowledging the limitations of the study, these findings suggest that the severity of the GNE mutations affects disease severity. The GNEM-DMP is a useful data collection tool, prospectively measuring the progression of GNE myopathy, which could play an important role in translational and clinical research and further understanding of genotype–phenotype correlations.

## Highlights

• Patient registry is a valuable tool in international GNE myopathy research.
• The registry expands the knowledge of GNE myopathy genetics and epidemiology.
• The registry allows monitoring of the disease progression and discovering diversity.
• The data suggest possible genotype–phenotype correlation in GNE myopathy.

## 1 Introduction

GNE myopathy has been originally described under several names: hereditary inclusion body myopathy (HIBM), distal myopathy with rimmed vacuoles (DMRV), quadriceps sparing myopathy (QSM) and Nonaka disease-reflecting clinical and biopsy presentation, genetic cause and history of discovery [1], [2]. It is a rare autosomal recessive myopathy caused by bi-allelic mutations in the *GNE* gene (UDP-N-acetyl-2-epimerase/N-acetylmannosamine kinase). Recently, GNE myopathy is the agreed term to name the condition, and the longest transcript of the *GNE* gene (hGNE2) is used for variant and mutation annotation [3].

The estimated prevalence varies between countries. The highest rates are reported in the Persian Jewish community [4], Japan (0.3), UK and Northern Ireland (0.44) [5] per 100,000, and in the Roma population in Bulgaria (incidence 1 in 500 births) [6]. Based on allele frequencies in three exome sequence databases, the global prevalence has been estimated at 0.4–2.1 per 100,000 [7].

The presentation and clinical course of GNE myopathy have been illustrated in several case reports and small cohort studies, most of them limited to a single centre or a particular country. The onset of GNE myopathy usually occurs in the third decade of life, but earlier and later appearances have been reported. It presents as a lower extremity distal myopathy that progresses proximally with relative sparing of the quadriceps muscle of the thigh. Upper limbs involvement occurs in parallel with the proximal leg musculature progression. The diagnosis is usually based on the pattern of muscle weakness, modest elevation of serum creatine kinase (CK) level, and a muscle biopsy showing rimmed vacuoles (on either H&E or Gomori's trichrome stain), and tubulofilamentous inclusions without evidence of inflammation [8]. Identification of mutations in both alleles of the *GNE* gene is critical for diagnostic confirmation of GNE myopathy. Several observational studies suggest that progression of the disease is steady and relatively slow, but ultimately leads to loss of ambulation within 10–20 years from the onset of first clinical symptoms [9], [10], [11], [12].

There is currently no approved treatment for this disease, but attempts are being made to supplement sialic acid deficiency with oral aceneuramic acid slow release [13] (www.clinicaltrials.gov ID number NCT02731690, NCT02736188, NCT01517880, NCT01830972, NCT01236898). Currently, a phase 3 study is underway (NCT02377921). Sialic acid precursor – N-acetyl-D-mannosamine (ManNAC) presents another promising therapeutic option. For the latter type of treatment, a Phase 1 trial (NCT01634750) is complete and a Phase 2 trial (NCT02346461) is currently ongoing). A single attempt of gene therapy in a patient was documented in 2011 [14].

Over 150 mutations scattered across the *GNE* gene have been reported to date [7]. Of these mutations, several were identified as founder or recurrent mutations [4], [5], [6], [15], [16], but many are sporadic and/or found in single families only.

Genotype–phenotype studies suggest that certain point mutations are linked to age at onset, presenting symptoms, severity and speed of the disease progression [10], [16]. The largest genotype–phenotype correlation study (n = 212) suggests that one of the most common mutations in Japan p. Asp207Val predisposes to later onset and milder phenotype as opposed to p.Val603Leu [9]. However, phenotype analysis in patients homozygous for the same mutation suggested significant inter- and intra- familial variability [6]. The Japanese study suggested phenotype difference between homozygous and compound heterozygous carriers [9]. No patient has so far been identified as carrying two nonsense or frameshifting mutations, suggesting that some basic activity of GNE is required during early development.

In this paper we describe the international online GNE myopathy Disease Monitoring Program (DMP) patient registry, which collects patient self-reported data from around the world. The registry collects medical and quality of life information reported by patients and is augmented by genetic and biopsy reports. The registry aims to contribute to a better recording of the disease course, its diversity and possible genotype–phenotype correlation.

## 2 Materials and methods

**Fig. 1:**
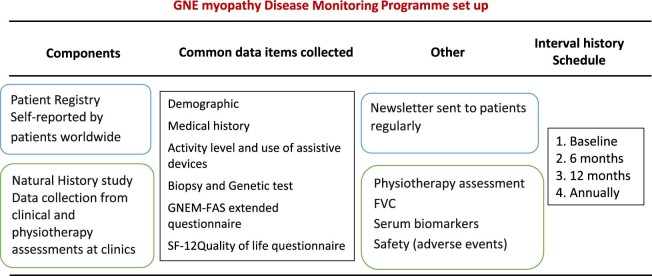
GNE Disease Monitoring Program study design. International patient self-reported registry run alongside the hospital based natural history study. Registry data set is governed by the Steering Committee and can be accessed via enquiries by the clinical and scientific community.

TREAT-NMD and Ultragenyx Pharmaceutical Inc. (Novato, CA, USA) established a public-private partnership in 2012 to conduct a GNE myopathy DMP linking two data collection efforts (Fig. 1). The study consists of two components: an international online registry with data entered by patients and a separate hospital based observational study with data entered by site staff per typical clinical research methods. The online DMP aims to acquire information on medical and diagnostic history, clinical symptoms and the progression of the disease from GNE myopathy patients worldwide. The study received full ethical approval from UK NRES Committee North East – Newcastle and North Tyneside 1 in 2013 (approval ID 13/NE/0123. ClinicalTrials.gov Identifier: NCT01784679). All participants have consented prior to their participation in the study.

### 2.1 Online registry design

The registry www.gnem-dmp.com was set up as a patient self-reported online database for patients clinically diagnosed or genetically confirmed with GNE myopathy. Patient participation is not restricted to any specific geographic location. All participants have been provided with patient information and a consent form. Patients were also encouraged to contact the registry curator at Newcastle University, their doctor or patient advocacy groups if they had any questions. Participants can sign the consent form electronically or use hard copies. The signed consent form allows the registry curator to contact the participant for future data validation, research and communication purposes. It also allows the registry curator to contact the doctor they nominate in order to request missing information and the validation of participant entered data. No protected health information is shared with the corporate sponsor. After informed consent has been obtained participants are asked to complete a set of validated 12-item short form survey (SF12), GNEM-FAS and non-validated questionnaires that are similar (where applicable), to the natural history study data set, to describe their medical history, level of activity, use of assistive devices and quality of life. Participants can upload their laboratory test results including muscle biopsies, genetic reports and general medical documentation to the registry. The registry has been online since 1 March 2014 and will continue to enrol and follow patients over time.

### 2.2 Interval history data collection

Patients are encouraged to log in to the registry at 6-month, 12-month and then annual intervals in order to update their profile and report any changes in their medical history, activity level or quality of life.

### 2.3 Database platform

The original database platform (1 March 2014–30 October 2015) was split between two vendors. Summit Analytical LLS (Denver, USA) holding de-identified participant data, and Orchid Inc. (Washington, USA) holding data, which is identifiable. The data was subsequently migrated to the current database platform, Digital Infuzion (Maryland, USA). The database platform consists of a participant facing front-end where consent and questionnaire forms are completed and a back-end, designed for use by the registry curator to review and manage entered data.

### 2.4 Patient recruitment

The registry was promoted via the dissemination of information on www.clinicaltrials.gov, neuromuscular and rare disease networks (e.g. TREAT-NMD, Orphanet) and patient organisations (e.g. Muscular Dystrophy UK (MD UK) and Neuromuscular Disease Foundation (NDF) (USA). Information about the registry was also included in genetic reports confirming GNE myopathy issued by the Northern Genetic service (UK). When registering, patients provide their names and consents for participation in the registry and for contact by registry curator with Newsletters and other relevant information.

### 2.5 Data management

The data collection follows EU data protection directive 95/46/EC. The registry is curated by TREAT-NMD. Data entered into the registry is stored securely on databases managed by Digital Infuzion, where access to the data set is limited to named individuals at Newcastle University and Digital Infuzion. Study principal investigator, registry curator and team involved in the platform development have access to the complete data set including identifiable data. No identifiable data are available to other study team members or to the corporate sponsor. The registry curator verifies all entered data via medical documentation provided by the participant, or alternatively by contacting the clinician nominated by the participant (upon participants' consent). Participants are able to withdraw their consent and entered data in the registry at any time.

### 2.6 Information governance and third party enquiries

A steering committee, with a defined charter and representation from public, private and academic sectors was established in 2014. It consists of seven members: 2 from TREAT-NMD, 2 international academic experts, 1 from Neuromuscular Disease Foundation, and 2 from Ultragenyx. The Steering Committee has strategic input and ensures that the registry is functioning in the patients' best interests. Third parties (e.g. researchers) may place an enquiry for access to the dataset to ask specific questions. The Steering Committee makes a decision about the request. In cases where the decision is positive, researchers may need to seek ethical and other relevant approvals, before receiving a report or other service from the registry.

### 2.7 Data analysis

The data analysed for this report was collected between 1 March 2014 and 1 October 2016. The data was checked for consistency and quality. Where necessary, the registry curator contacted patients to clarify entered data. The data entered in the registry by patients were augmented by the genetic and biopsy reports provided by the patients. Data analysis was performed using SPSS statistics version 22. Descriptive statistics and mean values with 95% CI are presented where applicable. Linear regression analysis, ANOVA, and Chi square test were used to test for an association among GNEM-FAS score, medical history parameters and genotype. Linear regression analysis was used to test for an association between GNEM-FAS score and duration of the disease.

## 3 Results

Between 1 March 2014 and 1 October 2016 (31 months) 269 participants registered with the www.gnem-dmp.com. The monthly rate was between 1 and 19 (average n = 7) registrations. Five newsletters were distributed to the patients. Newsletters supported the recruitment into a clinical trial, a natural history study, and two patient advocacy meetings. Newsletter recipients appreciated the information provided in their native language; they benefitted from specific content of the newsletters, related to medical and social aspects of the disease. Participants were very proactive in suggesting discussion points for the newsletters and in sharing their life stories. We have conducted a survey asking registry participants about their experience in completing the questionnaires, navigating the website and registry curator support. The survey showed that patients were satisfied with their experience. The newsletters were also distributed via the TREAT-NMD network and increased awareness among healthcare professionals. Several patient organisations and support groups were popularised via the newsletter and in turn provided articles and content to the newsletters.

The registry assisted with the identification of subjects in the GNE natural history study (NCT01784679) and Phase 3 clinical trial (NCT02377921). One hundred and fifty patients completed the questionnaires and were included in the analysis. The registry curator has followed up with all patients to clean the data and minimise the amount of missing data.

### 3.1 Baseline demographics

Registry participants represent 26 countries (Supplement Fig. S1). The following countries contributed the largest number of patients: Iran, Italy, South Korea, USA, UK, and India together represent 77.7% (n = 115) of all patients that answered questionnaires. Male and female participants constitute similar proportions (male n = 72, 48%, female n = 78, 52%). Mean age at baseline and body mass index (BMI) were as follows: male mean age = 38.7 [range 19–70 years], BMI = 25.9 kg/m^2^, female mean age =  39.5 [range 20–74 years], BMI = 23.5 kg/m^2^ Breakdown of age groups is shown in Supplement Fig. S2.

The breakdown of ethnical background: White n = 85 (56.6%); Asian n = 54 (36.6%), Black African n = 1 (0.7%), Mixed n = 4 (2.7%), “I do not want to disclose” n = 6 (4%). HIBM is the most commonly used name (58.7% of the cases), followed by GNE myopathy (25.4%), DMRV (8.7%), Nonaka disease (5.1%), LGMD (0.7%, confirmed GNE mutations). Fourteen families have several siblings (between 1 and 7) affected by GNE myopathy. One to three siblings from each family signed up to the registry. This constitutes 30% of all registry participants.

### 3.2 Onset, progression and diagnosis

Onset and first symptoms were recorded predominantly between 20 and 40 years (84.8%). Mean age of onset was at 27.8 years (95% CI 26.6–29.0. range 15–50 years old). Women had an earlier onset than men: female mean age = 26.5 years (95% CI 24.9–28.2), male mean age = 29.2 years (95%CI 27.5–30.1), p = 0.027. The earliest subclinical presentation was described as a “slow runner since 3 years old”. Early onset (<20 years) was observed in 11.4% of patients, late onset (>40 years) in 3.8%. The latest onset was observed in 1 patient at 50 years of age.

**Fig. 2:**
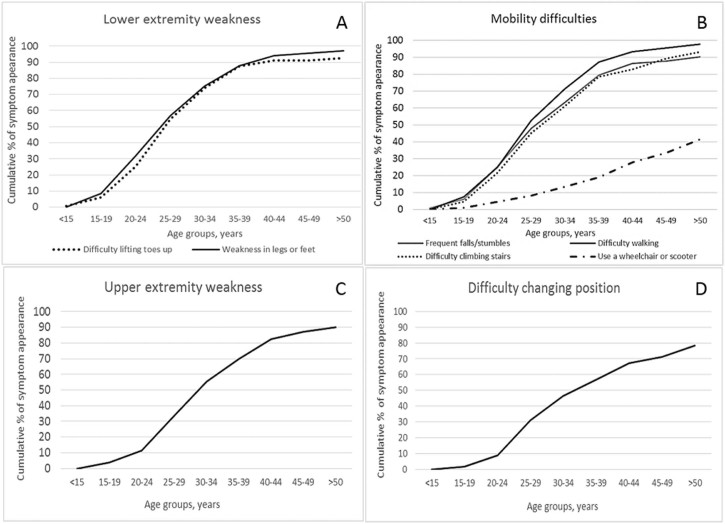
Cumulative percent of age of symptom appearance. A. Lower extremity difficulty. B. Mobility difficulties C. Upper extremity weakness D. Difficulty changing position.

Symptoms reflecting lower extremity weakness presented in most of the patients (Fig. 2a) (mean age 28.8 years). Difficulty lifting toes was experienced by 93.3%, weakness in legs and feet by 97.0%, difficulty walking by 97.8%, frequent falls or stumbles by 90.2%, difficulty climbing stairs by 93.2% of the respondents (Fig. 2b).

Weakness in arms and hands was reported in 90.3% of patients (Fig. 2c), on average starting 4 years later (32.8 years) than lower extremity symptoms. Difficulty sitting unaided (without any support) and changing position (i.e. turning in bed) were reported in 22.4% and 79.1% of respondents (Fig. 2d) at average age of 34.0 and 33.7 years respectively. Feeling fatigued was a common complaint (89.6%), as well as muscle spasms or twitching (59.7%) and muscle pain (45.9%).

### 3.3 Use of assistive devices and ambulation status

Non-ambulatory status was assigned to patients who cannot walk a short distance within the house and who use the wheelchair all the time. Non-ambulant patients at baseline constitute 24.3% of the registry population.

Ankle-foot orthoses (AFO) were used by 41% (n = 57) of respondents, a small number of patients were using high top boots 2.1% (n = 3) and shoe inserts 7.2% (n = 10). As displayed in Table 1, on average patients start to use AFOs at the age of 33.0 years (95% CI 30.8–35.3), a few years after the average age for first lower extremity symptoms.

**Table 1:** Estimated timeline of main milestones including disease onset and progression, and age at diagnosis.

Criteria	Mean years (SD)	Mean years since the onset (SD)
Age at onset	27.8 (7.0)	–
Lower extremity weakness	28.8 (6.7)	1.2 (3.6)
Age at diagnosis	32.7 (9.5)	5.2 (10.9)
Upper extremity weakness	32.8 (8.7)	5.2 (5.2)
Use of AFO	33.0 (7.7)	4.8 (5.7)
Difficulty sitting unaided	34.0 (9.0)	7.9 (5.6)
First use of wheelchair	38.3 (9.5)	11.9 (6.1)

Approximately 3 years later, at a mean age 36.3 years (95% CI 33.5–39.1) some patients (41%) start using assistive devices to help them walk. A cane was used by 22% of all respondents, crutch by 6.2%, and walker by 17.4%.

Wheelchair or scooters were used (36.4% of all respondents) by non-ambulant patients and by the weaker ambulant patients for longer journeys (e.g. travels through the airport or shopping). First use of a wheelchair was reported at a mean age of 38.3 years (95% CI 35.5–41.1). On average it took 11.9 years from the onset until a wheelchair is required (95% CI 10.2–13.5), [range 5–25 years].

### 3.4 Genotype analysis and genotype–phenotype observations

To calculate the age at diagnosis we used the date of the genetic report confirming *GNE* mutations. Mean age at diagnosis was 32.7 years (95% CI 31.0–34.5) in the subset of patients (n = 93) with available genetic reports. The time gap between the reported onset of the disease and diagnosis averaged 5.2 years (95% CI 3.0–7.4 years), [0–29 years].

**Fig. 3:**
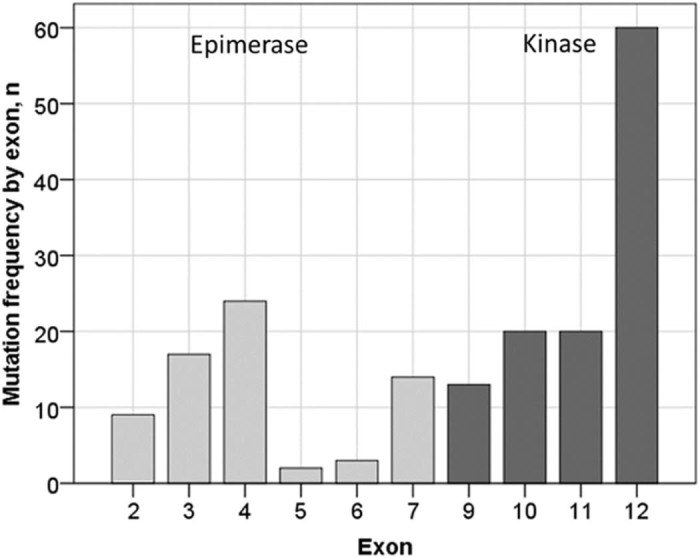
Mutation distribution by exon and kinase or epimerase domain.

Review of genetic reports showed that both nomenclatures (hGNE1 and hGNE2) are in use by the laboratories in various countries. Mutations in the *GNE* gene were detected in exons 2–12, but not in exon 1 and 8 (Fig. 3).

A wide range of mutations was identified. Most of the mutations (50/58) are missense; other mutations were: 1 nonsense, 1 frame shift, 3 deletions, 1 large deletion, 1 duplication and 1 deletion/insertion. Six of the most common mutations accounted for over 60% of all alleles observed in the registry population (Table 2). A complete list of mutations is shown in Table S1 of supplementary materials.

**Table 2:** List of most common mutations.

Nucleotide change	Amino acid change	Number of patients, n =	%	Most common country of origin	Country of origin (other)
c.2228T > C	p.Met743Thr	37	20.2	Iran	Saudi Arabia, Italy, USA
c.1985C>T	p.Ala662Val	19	10.4	UK	USA, Ireland
c.2179G>A	p.Val727Met	17	9.3	India	Bangladesh, UK, Germany, Guyana, USA
c.829C>T	p.Arg277Trp	16	8.7	Iran	Moldova, India, UK
c.1807G>C	p.Val603Leu	12	6.6	South Korea	
c.1225G>T	p.Asp409Tyr	9	4.9	UK	
Total		110	60.1		

Genotype–phenotype observations were analysed by domain and specific types of mutations as follows:

1. Patient reported outcomes were compared among three categories: A. two mutations in the epimerase domain, B. one mutation in the epimerase and kinase domain, C. two mutations in the kinase domain (Table 3).

**Table 3:** Genotype–phenotype correlation analysis. Baseline demographics and outcome factors analysed by the mutation location (epimerase, kinase or both). Also 4 most common mutations are included in the analysis.

	Fixed factors, cohort description			Outcome factors			
	Genotype	Age at baseline, mean (95% CI)	Sex, F%/M%	% of ambulant patients	Mean age of onset (95% CI)	Mean GNEM-FAS score, total % (95% CI)*	Mean age of first use of wheelchair
By domain	(A) Epimerase [exon 1–7], n = 18	40.2 (35.3–45.2)	72/28	82.3	29.2 (24.8–33.5)	51.1 (37.0–65.2)	44.5
	(B) One epimerase, one kinase, n = 30	39.8 (36.1–43.8)	50/50	70.0	26.2 (23.5–28.9)	52.1 (41.7–62.5)	38.6
	(C) Kinase [exon 8–12], n = 41	40.4 (36.7–44.1)	46/54	82.1	29.7 (27.7–31.7)	62.9 (55.6–70.2)	41.5
By mutation	p.Met743Thr n = 20 (homozygous- 85%)	40.4 (35.3–45.5)	40/60	84.2	27.8 (25.0–30.7)	61.4 (50.8–72.0)	43.2
	p.Ala662Val, n = 15 (homozygous- 33%)	48.1 (42.3–53.9)	67/33	60.0	30.8 (26.5–35.1)	41.8‡ (27.7–55.9)	43.1
	p.Val727Met, n = 12 (homozygous- 8%)	39.2 (33.2–45.1)	42/58	100	30.8 (26.9–34.6)	77.8 (69.0–86.6)	46
	p.Arg277Trp, n = 11 (homozygous- 64%)	39.3 (33.0–45.6)	73/27	80.0	29.2 (24.5–34.0)	53.0 (33.1–72.3)	42
	Other (not carrying mutations above), n = 23	35.3† (30.1–40.0)	48/52	82.6	25.7 (22.3–29.4)	56.3 (44.1–68.5)	33

* Three domains of FAS questionnaire. Score calculated as % normal functionality (100%), which means no limitations in the mobility, upper extremity and self-care function.

† Age at baseline statistically different between p.Ala662Val and “other” group, p = 0.003.

‡ ANOVA test of GNEM-FAS score between p.Ala662Val and p.Val727Met group p = 0.002.
2. Patient reported outcomes were compared between homozygous and heterozygous carriers of the 4 most common mutations (p.Met743Thr; p.Ala662Val; p.Val727Met; p.Arg277Trp) where number of patients was n > 15; Patients carrying two heterozygous mutations from the selected list of mutations were excluded from the analysis.

Selected outcome parameters include: age at onset, age at first wheelchair use, percent of non-ambulant patients in the analysed group, total GNEM-FAS score at baseline. The age distributions between all subgroups (by domain and by specific mutations) are comparable. However, the gender proportions vary substantially between subgroups (Table 3).

The difference in outcome measures between the groups was not statistically significant, but a trend was observed. Patients with one mutation in each domain (group B) had the lowest proportion of ambulant patients, earliest onset and earliest first use of wheelchair, compared to patients having both mutations in either epimerase or kinase domain.

Analysis of outcomes between specific genotypes is presented in Table 3 with statistically significant differences marked. There were a few general trends observed. Genotype p.Val727Met appears to have a milder phenotype with no non-ambulant patients to date, later onset, highest GNEM-FAS score and latest age of first wheelchair use.

Genotype p.Ala662Val group has the lowest proportion of ambulant patients and lowest GNEM-FAS score which might partially be explained by the fact that this group is the oldest in this data set.

Genotype p.Arg277Trp and p.Val727Met group have similar age at study baseline, however the p.Arg277Trp has 20.0% less ambulant patients and much lower (24.8%) GNEM-FAS score, which may indicate that this genotype predisposes to a more severe phenotype.

### 3.5 Muscle biopsy reports analysis

Most of the patients (86.9%) have had a muscle biopsy. Fifty-two biopsy reports were made available for review. Most common muscle biopsy sites were *Tibialis anterior, Biceps brachii, Deltoid, Quadriceps* (unspecified), *Gastrocnemius, Gluteus maximus, Rectus femoris, Peroneus longus*, hamstring (unspecified), and other unspecified muscles. The most common presentation is described as a myopathic or neurogenic pattern with rimmed vacuoles. The following findings of characteristics of a myopathy were observed: fibre size variation, fibre necrosis and regeneration and internalised nuclei. The following signs indicating a neurogenic component were observed: angulated fibres, clumped nuclei, and grouping of fibres. The abundance of rimmed vacuoles correlates with the stage of the disease (more vacuoles in more affected muscles) and the site of biopsy. The most affected muscle, e.g. *Tibialis anterior*, is affected at early stages of the disease and shows multiple rimmed vacuoles. *Quadriceps* however is spared until late stages and does not tend to exhibit rimmed vacuoles. Inflammatory findings and normal biopsy were noted on 2 and 1 occasions, respectively. Immunohistochemistry analysis showed no protein deficiency.

### 3.6 Longitudinal data analysis

**Fig. 4:**
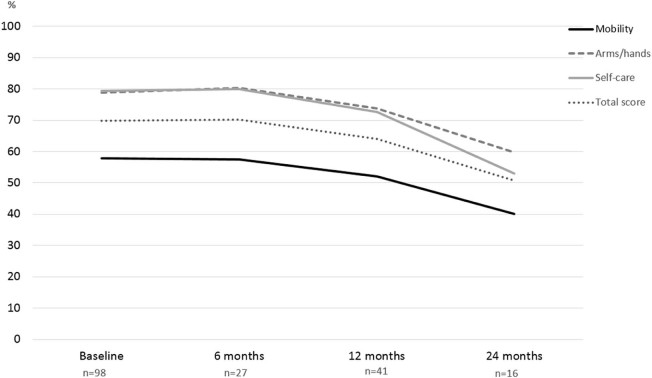
GNEM-FAS extended questionnaire analysis over 2 years in ambulant patients. Three domains of GNEM-FAS extended: mobility, upper extremity function and self-care analysed separately alongside the total FAS score.

**Fig. 5:**
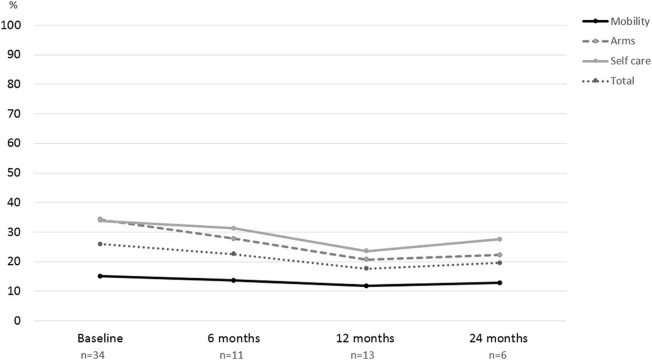
GNEM-FAS extended questionnaire analysis over 2 years in non-ambulant patients. Three domains of GNEM-FAS: mobility, upper extremity function and self-care analysed separately alongside the total FAS score.

To assess longitudinal changes we asked registry participants to complete medical history and GNEM-FAS questionnaires at 6-month, 12-month and 24-month time points. One patient (2%) became non-ambulant in the first 12 months of observation. The GNEM-FAS extended questionnaire (which includes extra questions appropriate for patients with significant mobility impairment) was analysed by domain: mobility, upper extremity, self-care and a total score (sum of scores in all three domains). The GNEM-FAS extended questionnaire has 30 questions, with answers rating from 0 (unable to do) to 4 (having no difficulty to do). Each score is weighted equally. The data is presented in the table in two forms: score and percent of a maximum achievable score (100% or normal functional level corresponds to 120 points on the total scale). Data from ambulant (Fig. 4) and non-ambulant (Fig. 5) patients were analysed separately due to the difference in physical ability. Clear functional decline in ambulant patient was observed at all three FAS domains over 2 years. The ability to move (e.g. walk, frequent falls or stumbles, weakness in legs) saw the greatest reduction in functionality, with on average 57.8% of normal functionality preserved. Over two years, the FAS score for the mobility domain dropped by 17.8%. Upper extremity function and self-care were both better preserved with 78.9% and 79.4% of normal function respectively at baseline. However, we observed a significant decline in both domains by 19.2% and 26.4% respectively over 2 years.

Non-ambulant patients were significantly weaker with 15% of mobility preserved. Similar to the ambulant group, upper extremity and self-care function were better preserved with 34.3 and 33.8%, respectively at baseline. There was a decline in function across all domains in the first year, however year 2 data analysis showed only a slight increase in FAS score across all 3 domains. Only 6 non-ambulant patients reported GNEM-FAS scores at the 24-month time point, so the small number of observations may potentially introduce bias. The speed of functional decline in ambulant patients was slower than in non-ambulant patients (Table 4 A, Table 4 B). Analysis of GNEM-FAS score in a subset of patients who completed all four visits over 2 years showed steady and statistically significant decline.

**Table 4 A:** Functional activity scale (FAS) analysis. FAS domains (mobility, upper extremity and self-care) are analysed in ambulant (A) and non-ambulant (B) cohorts separately. The data presented as mean score with 95% CI and mean % of maximum achievable score/function with 95% CI.

A Ambulant		Baseline, n = 98	6 months, n = 27	12 months, n = 41	24 months, n = 16
		Mean (95% CI)	Mean (95% CI)	Mean (95% CI)	Mean (95% CI)
Mobility	Score	30.0 (27.8–32.3)	29.9 (26.5–33.2)	27.1 (24.1–30.1)	20.8 (15.7–30.0)
	%	57.8 (53.5–62.0)	57.4 (50.9–63.9)	52.1 (46.3–57.9)	40.0 (30.1–50.0)
Upper extremity	Score	28.4 (27.0–29.7)	28.9 (26.6–31.2)	26.5 (24.1–29.0)	21.5 (16.7–26.4)
	%	78.9 (75.1–82.6)	80.3 (73.8–86.7)	73.7 (67.0–80.4)	59.7 (46.2–73.2)
Self-care	Score	25.4 (24.3–26.5)	25.6 (23.5–27.6)	23.4 (21.1–25.4)	18.6 (15.5–21.6)
	%	79.4 (76.0–82.8)	79.9 (73.5–86.3)	72.6 (66.0–79.3)	53.0 (48.4–67.6)
Total	Score	83.8 (79.7–88.0)	84.3 (78.1–90.5)	76.9 (70.6–83.1)	60.9 (49.8–72.0)
	%	69.9 (66.4–73.3)	70.3 (65.1–75.4)	64.1 (58.9–69.2)	50.7 (41.5–60.0)

**Table 4 B:** Functional activity scale (FAS) analysis. FAS domains (mobility, upper extremity and self-care) are analysed in ambulant (A) and non-ambulant (B) cohorts separately. The data presented as mean score with 95% CI and mean % of maximum achievable score/function with 95% CI.

B Non-Ambulant		Baseline, n = 34	6 months, n = 11	12 months, n = 13	24 months, n = 6
		Mean (95% CI)	Mean (95% CI)	Mean (95% CI)	Mean (95% CI)
Mobility	Score	7.9 (5.0–10.7)	7.1 (1.4–12.8)	6.1 (2.3–10.0)	6.7 (1.5–14.8)
	%	15.1 (9.6–20.6)	13.6 (2.7–24.6)	11.8 (4.4–19.3)	12.8 (2.8–28.4)
Upper extremity	Score	12.4 (9.4–15.4)	10.0 (4.3–15.7)	7.5 (3.2–11.7)	8.0 (2.1–13.9)
	%	34.3 (26.0–42.6)	27.8 (11.9–43.7)	20.7 (9.0–32.5)	22.2 (5.7–38.7)
Self-care	Score	10.8 (8.1–13.5)	10.0 (4.3–15.7)	7.5 (4.1–11.0)	8.8 (2.0–15.7)
	%	33.8 (25.3–42.3)	31.3 (13.5–49.0)	23.6 (12.8–34.3)	27.6 (6.3–48.9)
Total	Score	31.0 (22.8–39.2)	27.1 (10.6–43.6)	21.2 (10.4–31.9)	23.5 (3.7–43.3)
	%	25.9 (19.0–32.7)	22.6 (8.8–36.3)	17.6 (8.6–26.6)	19.6 (3.1–36.1)

**Fig. 6:**
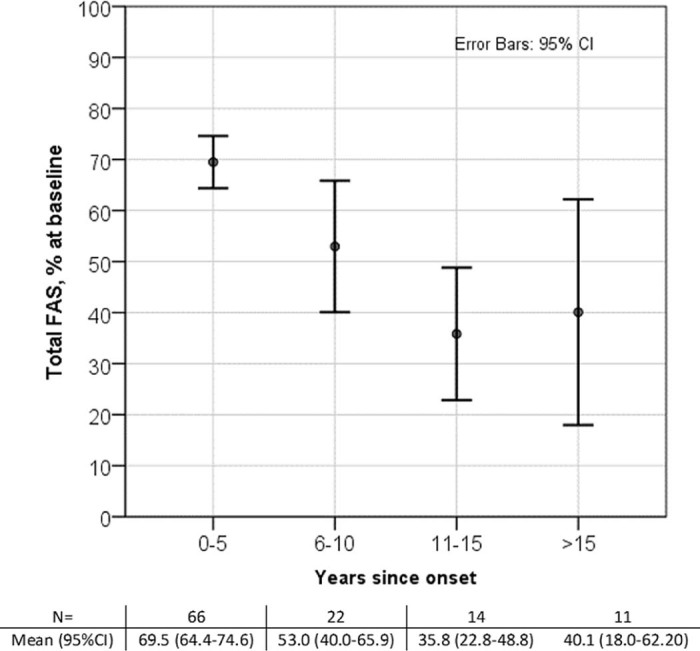
GNEM-FAS extended analysis by years since the onset.

The overview of the GNEM-FAS score in the whole cohort shows a linear negative correlation between duration of the symptomatic period and GNEM-FAS score (beta = −0.475, p < 0.001) (Fig. 6). A slight increase of GNEM-FAS score in a group of >15 years duration, also has higher CI, reflects a smaller sample size. As expected, patients with a longer history of the disease are weaker. This may suggest that patients with a longer duration of the disease, regardless of their actual age, are in a greater need of technical and social help in their daily activities and therefore have a greater impact on costs of disease health economics.

### 3.7 Other concomitant conditions and diseases

#### 3.7.1 Depression

Patients with clinically diagnosed depression and those receiving treatment for depression, totalled 9.5% (13/137) of registry participants included in analysis. Based on the SF12 questionnaire, and health and wellbeing questionnaires, 13.0 to 19.3% of respondents feel low and depressed most or all of the time respectively.

#### 3.7.2 Pain

A significant number of patients experienced musculoskeletal pain (45.9%). The most common location of pain was legs (32.6% of all respondents), then arms and hands (10.5%), back (9.8%), shoulders (9.0%), and neck (4.5%).

#### 3.7.3 Cardiovascular and respiratory involvement

In medical history analysis we focused on conditions related to the cardiovascular and respiratory system.

Thirteen respondents (9.5%) reported that they have a cardiovascular condition. The diagnosis was reported as follows; cardiomyopathy (treated with medication) – 1.5% (2/137), high blood pressure – 5.8%, palpitations and tachyarrhythmia – 1.5%, deep vein thrombosis – 4.5%, on warfarin treatment (unspecified) – 0.7%, mildly impaired left ventricular systolic function – 0.7%.

Respiratory difficulties (“difficulty breathing”) were noted by 10.2% (n = 14). Four patients have asthma (2.9%), in 4 patients difficulty in breathing was associated with cardiovascular conditions (arrhythmia, palpitations, HBP, and cardiomyopathy) and 2 of them use CPAP for an unspecified reason. Four other patients reported breathing difficulties due to anxiety (n = 1) or other unspecified reasons (n = 3).

## 4 Discussion

Rare and ultra-rare diseases often reveal gaps in understanding disease diversity, its characteristics and disease modifying factors due to the small number of observed patients. Most of the knowledge currently available on GNE myopathy are derived from case reports or local (they were not too small compared to this study) cohort observations. Patient registries, although subject to the natural bias of patient-reported data, have proven useful in longitudinal observational data collection, establishing a pool of patients for clinical trial readiness and contributing to an overall better understanding of rare diseases [17], [18]. Specifically, the Japanese national GNE registry Remudy has provided valuable insights into the demographic, medical history and correlation between phenotype and genotype on the largest number of GNE myopathy patients in one country [19], [20]. To our knowledge, there are no other national GNE specific registries.

Rare diseases impose specific challenges in clinical research due to small sample size and heterogeneity of the disease course. Therefore, clinical research into rare diseases including GNE myopathy often requires coordinated, multicentre, international collaboration in order to combine effort and knowledge under one roof in a standardised process. We have addressed these challenges by setting up a centralised, international registry as part of the GNE myopathy Disease Monitoring Program (DMP). The private-public partnership coinciding with the oversight from the international Steering Committee has successfully ensured meaningful, secure and sustainable data collection via the online registry platform.

The registry or online portion of the GNEM-DMP is a valuable tool in delivering relevant medical and scientific information to the participants. Newsletters translated in to a wide range of languages have provided an opportunity to engage with participants internationally, giving them the opportunity to also participate by writing their own articles for inclusion. The registry has also assisted in recruitment to clinical research activities, surveys and patient advocacy meetings.

Genetic basis for GNE myopathy was established relatively recently with the causative gene discovered in 2001 [21]. Geographical distribution of the cases in the GNE registry shows the global representation of GNE myopathy with similar proportion of men and women affected. However, the women enrolled in the registry tend to have an earlier onset on average than men, which has not been previously reported. A possible explanation could be that there is an aggravating effect of pregnancy on muscle strength in general and possibly by increased requirement of Sialic acid during pregnancy [22].

Although GNE myopathy has a distinct clinical presentation, it is important to confirm the diagnosis genetically and to exclude other distal myopathies with similar presentation for a correct care and disease management. Registry participants reported on a wide range of diagnostic delays between onset of the disease and genetic confirmation (between months and decades). Recently, GNE genetic testing became more widely available which reduced the waiting times for patients.

The presented timeline of the disease milestones confirms the slow, but progressive course and a noticeable variability between patients even with the same genotype [8], [9], [23], [24].

The early requirement for the use of orthotics may reflect the specific pattern of muscle weakness, e.g. distal leg involvement (foot drop). Individual progression from the onset of first symptoms to the wheelchair use stage is variable and could be linked to specific GNE genotype and other yet undiscovered modifiers. Also it may represent the local financial support for wheelchair supply.

**Fig. 7:**
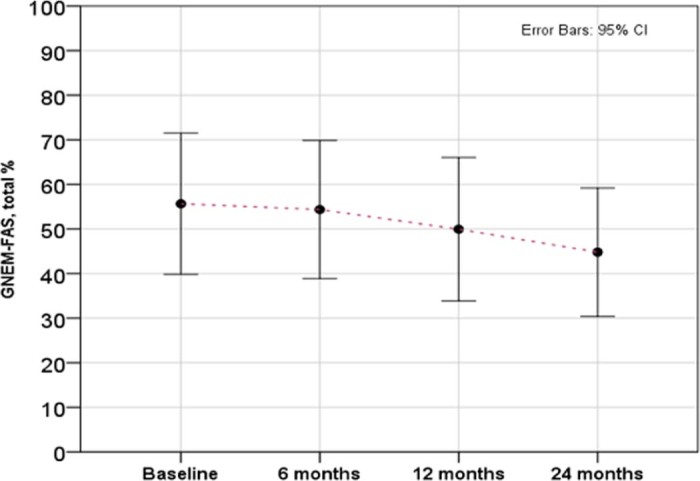
Steady decline of GNEM-FAS score in a subset of patient who completed all four interval history visits over 2 years (n = 16). 24-month score is significantly lower compared to baseline, ANOVA, p = 0.016.

To measure the actual decline of the function we used the patient reported outcome measure- the GNEM-FAS questionnaire, specifically designed for GNE myopathy and used in clinical research and trials [25]. For this report, measurable decline was determined at 4 time points over 2 years in ambulant and non-ambulant cohorts (Fig. 7). The most affected item is mobility and the most preserved is upper extremity function. This is consistent with a natural history study conducted in Japan in ambulant and non-ambulant patients, where physical outcome measures were used [26].

The decline over 2 years was similar across three GNEM-FAS domains in ambulant patients. Non-ambulant patients have much lower baseline GNEM-FAS score and slower decline of the remaining muscle function, however measurable decline was also observed and could serve as an important baseline marker for efficacy assessments of the new therapies. This is in line with the natural history studies [26], [27].

Observed relationships between genotype and self-reported clinical parameters indicate that the severity of the disease might be partially attributable to the specific *GNE* genotype. In our study, patients with bi-allelic mutations in both the epimerase and kinase domains were predisposed to a more severe phenotype compared to the patients with both mutations located in just one domain. This could partially be attributable to the fact that mutation that is expected to cause more severe phenotype was prevalent in the epimerase and kinase groups. This observation is in contrast with findings in 35 Chinese patients with GNE myopathy, which suggested that mutations in the epimerase domain may lead to atypical clinical presentation and later onset compared to the patients with both mutations in the kinase domain [16]. In that cohort, Chinese patients carried the milder D207V mutation. As a result, the domain-specific analysis could be obscured by the mutations. Moreover, this study had a smaller sample size from one ethnic group only; therefore the results are not fully comparable and may not easily be extrapolated to other ethnicities and countries. The molecular mechanisms underlying genotype–phenotype correlations are yet to be elucidated and may involve a GNE protein function other than sialic acid synthesis.

Limitations in our study include reliance on patient reported information and potential for bias given the voluntary nature of patient's participation in the GNEM-DMP on-line registry.

Our data taken together with a literature review suggest that within one domain, GNE mutations could result in a severe and mild mutations depending on the dominating mutation in the analysed cohort.

Detailed analysis of individual, highly prevalent mutations showed that the mutation p.Ala662Val (UK) may predispose to a more severe phenotype than the mutation p.Val727Met (India) p.Ala662Val demonstrating with an earlier onset and faster progression of the symptoms. Similarly, a difference between two Japanese founder mutations was previously described [24]. Comparative analysis of enzyme activity in heterologous systems modelling different GNE mutations supports the hypothesis that the mutations are not identical in their effect on protein stability and enzyme activity [28]. Therefore, it is important to consider individual trajectories and potential genotype–phenotype correlations for patient stratification in clinical trials and for clinical management.

GNE myopathy does not appear to be associated with an increased risk of cardiomyopathy, CVD or respiratory failure [12], however respiratory difficulty is observed in advanced stages of the disease [24]. Our initial data do not suggest an increased risk of respiratory difficulty, in GNE patients in comparison to the general adult population. Dilated cardiomyopathy and cardiac conduction abnormalities, and sudden death have been reported in GNE myopathy occasionally [29], [30]. In the GNEM-DMP registry dataset several cases of cardiac conduction abnormalities, cardiomyopathy and respiratory difficulty (including use of CPAP) were identified. The rate of cardiomyopathy in the registry data set is low (2/137), but formally higher than in general adult population (1:2500 [31]). However, considering the low number of observations in the registry and low statistical power of the analysis we cannot conclude whether the risk of cardiomyopathy in GNE patients truly increased.

## 5 Conclusions

In this initial report from the GNE myopathy DMP-registry, we have illustrated the successful implementation of an international registry based on a public-private partnership model and its versatile use as both a tool for disseminating information and also for recruitment to clinical trials. Data showing the disease presentation and progression support previous, retrospective reports of GNE myopathy as a slowly progressive disease and contribute to a step-by-step understanding of the disease progression, and also allow to quantify and time-stamp these changes. Patient reported outcomes used in this study showed the longitudinal decline in mobility, upper extremity function and self-care and may help to power future studies and support the development of management guidelines as well as socio-economic assessments (burden of illness studies). The observed relationships between phenotype and genotype may help to predict progression of the disease and support patient stratification in clinical trials depending upon individual rates of disease progression. Study limitations such as restrictions of the data collection technique, small sample size (and therefore low statistical power), confounding factors (e.g. age at baseline, and gender) allow us to report these findings as an initial observation, which needs to be replicated on a larger cohort before conclusions can be drawn. This is a first report based on the international GNE myopathy registry, which continues to evolve and collect data on a large population over a long period of time. To conclude, the registry is an effective method of data collection and information dissemination, which can contribute valuable knowledge and address scientific difficulties in a small and multi-ethnic cohort and help translational research in rare disease.

## Appendix Supplementary material

Appendix S1 Figure S1 and S2. Appendix S1

Table S1 List of mutations in registry participants. Table S1
