# Supplementary material for: Phenotypic stratification and genotype–phenotype correlation in a heterogeneous, international cohort of GNE myopathy patients: First report from the GNE myopathy Disease Monitoring Program, registry portion
Source: Neuromuscul Disord. 2018 Feb;28(2):158–68. doi: 10.1016/j.nmd.2017.11.001 (PMC5857291; doi:10.1016/j.nmd.2017.11.001)
Supplement: Appendix S1 — Figure S1 and S2. [file mmc1.docx]

**Supplement materials**


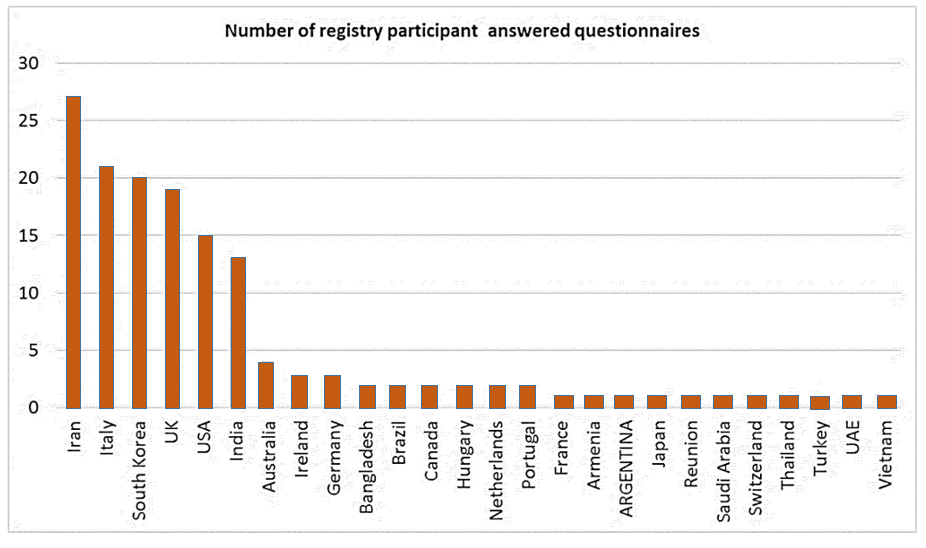


Figure 1. Number of patients registered with the GNE-DMP registry and completed questionnaires between 1 March 2014 and 1 November 2016.


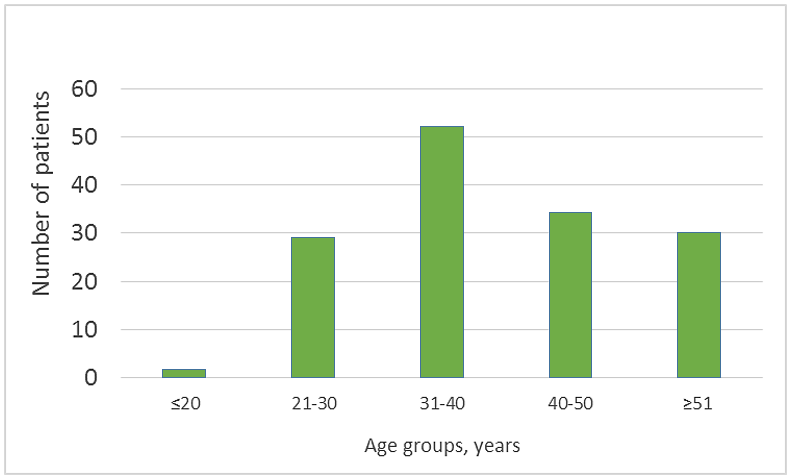


Figure 2 .Age at baseline by decade
