# Supplementary material for: Phenotypic stratification and genotype–phenotype correlation in a heterogeneous, international cohort of GNE myopathy patients: First report from the GNE myopathy Disease Monitoring Program, registry portion
Source: Neuromuscul Disord. 2018 Feb;28(2):158–68. doi: 10.1016/j.nmd.2017.11.001 (PMC5857291; doi:10.1016/j.nmd.2017.11.001)
Supplement: Table S1 — List of mutations in registry participants. [file mmc2.docx]

**Supplementary materials**

Table 1. List of mutations in registry participants.

^1^ Mutations not listed in Leiden database ([www.databases.lovd.nl/shared/genes/GNE](http://www.databases.lovd.nl/shared/genes/GNE))

| Nucleotide change | AA change | Exon |
| --- | --- | --- |
| c.124C>T | p.Arg42Trp | 2 |
| c.131G>C | p.Cys44Ser | 2 |
| ^1^c.191*FS5 | p.Glu64fs*5 | 2 |
| c.271A>G | p.Met91Val | 3 |
| ^1^c.331G>T | p.Asp111Tyr | 3 |
| ^1^c.340G>A | p.Ala114Thr | 3 |
| ^1^c.368G>A | p.Arg129Gln | 3 |
| ^1^c.400A>T | p.Lys134Asn | 3 |
| c.478C>T | p.Arg160* | 3 |
| c.479G>A | p.Arg160Gln | 3 |
| c.559T>C | p.Tyr187His | 3 |
| c.577C>T | p.Arg193Cys | 3 |
| c.620A>T | p.Asp207Val | 3 |
| ^1^c650A>G | p.Tyr217Cys | 3 |
| c.691A>T | p.Ile231Phe | 3 |
| ^1^c.705_706delinsCT | p.Trp235Cys | 3 |
| c.705G>A | p.Trp235* | 3 |
| c.709G>A | p.Gly237Ser | 3 |
| c.740T>C | p.Val247Ala | 4 |
| c.766G>A | p.Asp256Asn | 4 |
| ^1^c.805delG | p.Asp269Metfs*11 | 4 |
| ^1^c.809T>C | p.Ile270Thr | 4 |
| c.829C>T | p.Arg277Trp | 4 |
| c.830G>A | p.Arg277Gln | 4 |
| c.986T>C | p.Ile329Thr | 5 |
| ^1^c.992A>C | p.Asn331Lys | 5 |
| c.1096C>T | p.Arg366Trp | 6 |
| ^1^c.1129G>T | p.Val377Met | 6 |
| ^1^c.1178G>A | p.Gly393Glu | 7 |
| c.1223delT | p.Ile408fs*16 | 7 |
| c.1225G>T | p.Asp409Tyr | 7 |
| ^1^c.1285C>T | p.Gln429* | 7 |
| ^1^c.1313dupT | p.Ser439fs | 7 |
| ^1^c.1525C>T | p.His509Tyr | 9 |
| ^1^c.1610T>A | p.Phe568Ile | 9 |
| c.1618C>T | p.His540Tyr | 9 |
| c.1649A>G | p.Asn550Ser | 9 |
| c.1664C>T | p.Ala555Val | 9 |
| c.1676T>G | p.Phe559Cys | 9 |
| ^1^c.1705C>A | p.His569Asn | 9 |
| ^1^c.1748A>G | p.His552Arg | 10 |
| c.1760T>C | p.Ile587Thr | 10 |
| c.1807G>C | p.Val603Leu | 10 |
| c.1853T>C | p.Ile618Thr | 10 |
| c.1864G>A | p.Ala622Thr | 10 |
| ^1^c1909G>A | p.Lys633Asn | 10 |
| ^1^c1913 | p.G638Efs9X | 10 |
| ^1^c.1918C>T | p.Leu640Phe | 11 |
| c.1985C>T | p.Ala662Val | 11 |
| ^1^c.2028G>A | p.Gly700Arg | 12 |
| c.2116T>C | p.Tyr706His | 12 |
| c.2179G>A | p.Val727Met | 12 |
| c.2228T>C | p.Met743Thr | 12 |
| Deletion 1-9 |  | 1to9 |
